# Kidney-Detrimental Factors and Estimated Glomerular Filtration Rate in Preterm Newborns: The Role of Nutrition

**DOI:** 10.3390/nu12030651

**Published:** 2020-02-28

**Authors:** Alice Monzani, Ilaria Crespi, Giulia Genoni, Alberto Edefonti, Giovanni Montini, Giorgio Bellomo, Federica Ferrero, Simonetta Bellone, Flavia Prodam

**Affiliations:** 1 Division of Pediatrics, Department of Health Sciences, University of Piemonte Orientale, 28100 Novara, Italy; alice.monzani@gmail.com (A.M.); simonetta.bellone@med.uniupo.it (S.B.); 2 Clinical Chemistry Laboratory, Department of Health Sciences, University of Piemonte Orientale, 28100 Novara, Italy; ilaria.crespi@maggioreosp.novara.it (I.C.); bellomo.giorgio@gmail.com (G.B.); 3 Pediatric and Neonatal Intensive Care Unit, Maggiore della Carità University Hospital, 28100 Novara, Italy; federicaferrero@libero.it; 4 Pediatric Nephrology, Dialysis and Transplant Unit, Fondazione Ca’ Granda IRCCS, Ospedale Maggiore Policlinico, 20122 Milan, Italy; aedefonti@hotmail.com (A.E.); giovanni.montini@unimi.it (G.M.); 5 Department of Clinical Sciences and Community Health, Università degli Studi di Milano, 20122 Milan, Italy; 6 Interdisciplinary Research Center of Autoimmune and Allergic Diseases, University of Piemonte Orientale, 28100 Novara, Italy; flavia.prodam@med.uniupo.it; 7 Department of Health Sciences, University of Piemonte Orientale, 28100 Novara, Italy

**Keywords:** preterm newborn, kidney function, Cystatin C, Beta-trace protein, chronic kidney disease, parenteral nutrition

## Abstract

Background: Kidney function in preterm newborns may be impaired by many factors. Methods: 71 newborns with gestational age (GA) < 32 weeks were enrolled. Serum creatinine (sCr), cystatin C (CysC), beta-trace protein (BTP) and urea were measured at T0 (3rd day of life) and T36 (GA 36 weeks), and estimated glomerular filtration rate (eGFR) was calculated according to different formulas at T36. Pre-natal and post-natal kidney injury risk scores were calculated. Results: Newborns with GA ≤ 28 weeks had higher sCr at T0, and lower sCr, BTP and higher urea levels at T36 (*p* = 0.007, *p* = 0.005 and *p* = 0.029, respectively). eGFR values were not different according to GA when calculated by the formulas using only CysC, but were higher in subjects with GA ≤ 28 weeks according to the other formulas. The post-natal score was positively correlated with eGFR according to sCr-based formulas, but the correlations did not persist when adjusted for urea levels and GA. Conclusions: CysC-based eGFR values are not influenced by GA. Post-natal score shows a direct correlation with eGFR according to sCr-based formulas, not persisting after adjustment for GA and urea levels, implying the importance of the nutritional status, since more premature subjects receive a more aggressive nutritional regimen, testified by higher urea levels.

## 1. Introduction

The survival of preterm newborns has dramatically improved in the recent decades, with babies born as young as 25-week gestation having up to the 80% chance of survival [1]. Preterm birth has a potential detrimental influence on kidneys developmental programming. Nephrogenesis is completed between 32 and 36 weeks of gestation, with most nephrons being formed during the third trimester of pregnancy [2]. Therefore, preterm birth may negatively impact on nephron endowment. Additionally, any further impact on nephron number and function at the very beginning of life (nephrotoxic medications, mechanical ventilation with possible hyperoxia or hypoxia, hemodynamic instability) increases the risk of adverse consequences for life-long renal health [3].

In clinical routine, the most used endogenous biomarker for the assessment of neonatal kidney function is serum creatinine (sCr). However, in childhood it is dependent on age, gender and muscle mass. Furthermore, in premature newborns sCr shows a great variability in the first week of life in part due to the passage of maternal Cr through the placenta and in part to its reabsorption in the proximal tubules caused by tubular membrane immaturity, thus it is quite insensitive to detecting mild to moderate reductions in glomerular filtration rate. In the last years, new potential endogenous biomarkers have been proposed, such as cystatin C (CysC) [4] and beta-trace protein (BTP) [5]. CysC is a low molecular weight protein (13,343 Da, 120 amino acids) belonging to the cystatin superfamily of reversible inhibitors of cysteine proteases of the papain and legumain families [6]. In contrast with sCr, CysC does not seem to be affected by body muscle mass, age, gender, inflammatory state, or nutritional conditions [7]. As for creatinine, CysC is freely filtered through glomeruli. There are limited studies of CysC use to assess renal function in neonates [8,9,10], but it could be a promising biomarker as only minimal amounts cross the placenta, thereby reflecting mostly neonatal function [11]. Also BTP, a 23- to 29-kD enzyme consisting of 168 amino acids, may be a good marker of neonatal renal function because it is freely filtered at glomerular level and it does not cross the placenta at all [12].

In the present study we aim to evaluate CysC and BTP levels in preterm newborns, and to compare different formulas for estimating glomerular filtration rate (eGFR) based on CysC, BTP or sCr. Moreover, we aim to assess the impact of pre-natal and post-natal potentially kidney-detrimental factors on kidney function of preterm newborns.

## 2. Methods

We performed an observational longitudinal study in the Neonatal Intensive Care Unit (NICU) of Maggiore della Carità University Hospital, Novara, a tertiary level perinatal center. We enrolled all the preterm babies with a gestational age (GA) ≤ 32 weeks born between September 2015 and December 2017. Exclusion criteria were the presence of prenatally detected kidney anomalies and complex syndromes or congenital anomalies. The protocol was conducted in accordance with the declaration of Helsinki and was approved by the Local Ethic Committee of Maggiore della Carità University Hospital of Novara (CE 79/18). Written informed consent was obtained by the newborns’ parents.

Preterm babies were enrolled at birth and assessments were performed at two time points: T0, on the 3rd day of life, and T36, when they reached a GA of 36 weeks. The T0 timing was chosen on the basis that neonatal sCr values are influenced by maternal ones in the first 48–72 h after birth; the T36 timing because nephrogenesis is supposed to be completed at 36 weeks GA. Babies who were discharged or transferred to other hospitals or died before 36 weeks GA performed only the T0 assessment. 

### 2.1. Anamnestic and Clinical Course Data

From medical records, we collected for each subject the following data: gender, GA, birth weight, single/multiple gestation, delivery mode, use of nephrotoxic drugs during pregnancy, positive family history for arterial hypertension (within the second degree of kinship), history of intrauterine growth restriction (IUGR) or maternal pre-eclampsia/eclampsia. GA was determined by fetal ultrasound and last menstrual period. INeS charts [13] were used to evaluate birth weight categories. Nephrotoxic drugs administered to the mothers included betamethasone [14], non-steroidal anti-inflammatory drugs [15], anti-hypertensive drugs [16], and anti-microbial agents [17].

From our NICU dataset, we collected for each subject the following data: invasive and non-invasive ventilation and their duration, length of O2-support, bronchopulmonary dysplasia (BPD), sepsis, use and duration of antibiotic therapy especially nephrotoxic drugs (gentamicin, vancomycin), patent ductus arteriosus (PDA), pharmacological (ibuprofen) or surgical treatment for hemodynamic significant PDA, necrotizing enterocolitis (NEC), intra-ventricular hemorrhage (IVH), length of stay in the NICU, administration of parenteral nutrition and its duration.

The durations were expressed as number of days. The Yes/No items were used to create one prenatal and one postnatal kidney injury risk score (Yes = 1 point; No = 0), given by the sum of all the “Yes” answers to the presence of potential risk factors for kidney injury [16,17] (Table 1).

### 2.2. Nutritional Regimen

In the subjects receiving parenteral nutrition, the protocol was as follows: (i) total parenteral nutrition with a high level of amino acids (3 g/kg/day) from the first hours of life, (ii) lipids from the first 24 h of life at 1.5–2.5 g/kg/day increasing to 3.5 g/kg/day and (iii) minimal enteral feeding at 10–20 mL/kg/day from the first one to two days of life [18]. The parenteral solutions supplied a minimum of 51 kcal/kg/day with 3 g/kg/day of proteins on the first day of life, up to a minimum of 100 kcal/kg/day and 4 g/kg/day of proteins in the first week. Enteral feeding was initiated within 48 h of postnatal life using breastmilk or preterm formula when breastmilk was not available, and this provided 83 kcal/100 mL of energy. Enteral feeding stages were based on corrected age and on the clinical conditions of each infant. When the infants tolerated an enteral intake of ≥100 mL/kg, parenteral nutrition was discontinued.

### 2.3. Anthropometric Measurements

Weight and length were measured at T0 and T36. Birth weight was measured using an electronic weighting scale integrated into the incubator until the babies were taken in the incubators, and then by an electronic infant weighting scale. The crown-heel length measurement was carried out with a Harpenden neonatometer with the baby lying supine and with the body, hips, and knees straightened; measurements were taken twice and then averaged. Body surface area (BSA) was calculated according to Haycock formula:

BSA (m^2^) = 0.024265 × L (cm) 0.3964 × weight (kg) 0.5378

### 2.4. Biochemical Parameters

Venous or arterial blood samples were collected at T0 and T36, and sCr, CysC, BTP and urea were measured.

sCr: was measured on an ADVIA Chemistry 1800 or ADVIA Chemistry XPT with an enzymatic assay. This method is considered more specific and reliable than all the Jaffé modified methods. The analytical variability coefficient was <3%.

Cys C: was measured with BN II/ BN ProSpec^®^ by amplified latex immunophelometry. 

BTP: was measured on serum sample with N Latex BTP system. N Latex BTP is BTP specific. 

Urea: was measured on ADVIA Chemistry 1800 or ADVIA Chemistry XPT using the glutamate dehydrogenase lined enzyme assay system. The analytical variability coefficient was <2.5%.

eGFR was calculated according to 9 existing formulas, including sCr in 2 cases, CysC in 5 cases, BTP in 1 case and combining sCr and CysC in 1 case. The applied formulas were: (1) eGFR Schwartz 2009: 0.413 × lenght/sCr [19]; (2) eGFR Brion: 0.33 × lenght/sCr [20]; (3) eGFR Schwartz 2012: 70.69 × (CysC) ^−0.931^ [21]; (4) eGFR Zappitelli: 75.94 × (CysC) ^−1.170^ [22]; (5) eGFR Filler: 91.62 × (CysC) ^−1.123^ [23]; (6) eGFR Dorum: 74.835 × (CysC) ^−0.750^ [24]; (7) eGFR Treiber: [(TKV/BSA)/CysC]/1.73 [25]; (8) eGFR Benlamri: 10 ^(1.902+(0.9515 × LOG(1/BTP)))^ [26]; (9) eGFR Zappitelli-combined: (43.82 × *e* ^0.003 × L^)/(CysC ^0.635^ × sCr ^0.547^) [22].

### 2.5. Kidney Ultrasonography

At T0 and T36 a kidney ultrasonography (US) was performed by a 5–8 MHz sector transducer (Philips HD7XE Ultrasound System). To avoid inter-observer bias during scanning and measuring, all US scans of the kidneys were performed by a single neonatologist, skilled in renal US. Renal length, anteroposterior diameter (width), and transverse diameter (depth) were measured for both kidneys. Kidney volume (KV; ml) was calculated by the equation for an ellipsoid:

volume = length × width × depth × π/6.

Total kidney volume (TKV) was calculated by the sum of left and right kidney volumes, and its ratio to BSA was calculated too.

### 2.6. Statistical Analysis

Data were expressed as mean ± standard deviation (SD), for continuous variables, and as number and percentage (%), for categorical variables. 

For some analyses, subjects were divided into two subgroups according to GA (≤ and >28 weeks). Comparisons between groups were performed using Kruskal–Wallis analysis of variance (ANOVA) and the Mann–Whitney U test, as appropriate. Comparisons between paired data (T0 vs. T36) were performed by Wilcoxon signed rank test. Correlations between continuous variables were evaluated by Spearman rank order correlation. A p value of < 0.05 was considered statistically significant. All analyses were performed using SPSS version 21.0 (IBM, New York, NY, USA).

## 3. Results

At T0, we enrolled 71 newborns (M:F = 37:34), born between 24 and 32 weeks of GA (mean GA 28.5 ± 2.16). Out of them, 31 (43.7%) had a GA ≤ 28 weeks. At T36, data could be collected for 53 patients, since 5 newborns (7%) were dead and 13 (18.3%) were back-transferred before 36 weeks GA to other second-level neonatology units.

### 3.1. Anamnestic and Clinical Course Data

The delivery mode was vaginal eutocic in 13 newborns (19.4%), vaginal dystocic in 1 (1.5%), and caesarean section in 53 (79.1%). Sixty-one neonates were singletons (85.9%) and 10 were twins (14.1%). A history of IUGR was reported in 12 neonates (16.9%), of pre-eclampsia in 20 (28.2%), administration of at least one potential nephrotoxic drug in 54 mothers (76.1%). In 25 newborns (35.2%) a positive family history for arterial hypertension was reported. Seven newborns (9.9%) were classified as small for gestational age. 

Clinical course data are shown in Table 2. In the overall group, the mean prenatal and postnatal scores were 1.5 ± 0.9 and 3.6 ± 1.6, respectively. Comparing the groups of subjects with a GA ≤ 28 weeks and >28 weeks, the prenatal score was similar (1.4 ± 0.9 vs. 1.6 ± 0.9, NS), whereas the postnatal score was significantly higher in the subjects with a lower GA (4.5 ± 1.6 vs. 2.9 ± 1.3, *p* < 0.0001).

All the infants with a GA ≤ 28 weeks received parenteral nutrition, whereas 53.6% of those with a GA > 28 weeks did (*p* < 0.0001). Also duration of parenteral nutrition was significantly higher in newborns with a GA ≤ 28 weeks (24.6 ± 13.4 days vs. 15.7 ± 4.2, *p* = 0.02).

### 3.2. Anthropometric Data

Anthropometric data at T0 and T36 are shown in Table 3. At T0, all anthropometric parameters were significantly lower in newborns with a GA ≤ 28 weeks (*p* < 0.0001 for each), whereas at T36 they were not different in the two groups. No differences were found in any of the anthropometric measures according to gender at T0, whereas at T36 males had higher weight, length and BSA (*p* = 0.03 for each).

### 3.3. Biochemical and Ultrasonography (US) Data

Mean sCr, CysC, BPT, and urea values in the overall group and according to GA at T0 and T36 are reported in Table 4. 

**Figure 1:**
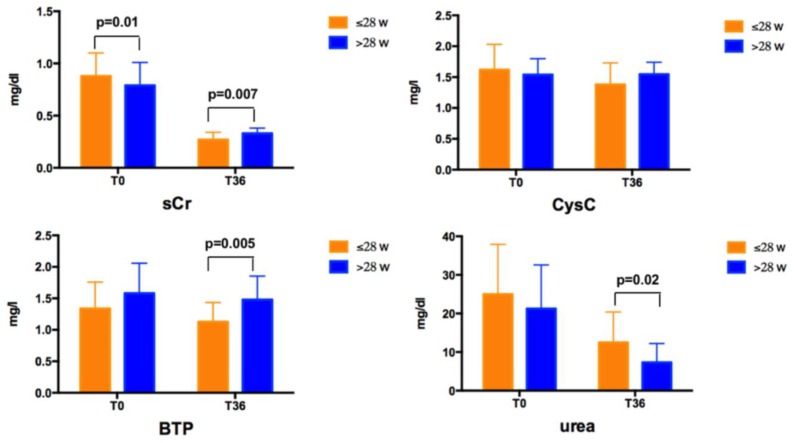
Serum Creatinine (sCr), Cystatine C (CysC), Beta-Trace Protein (BTP) and urea levels at T0 and T36, according to gestational age.

No differences were found for any of the biochemical and US data according to gender at T0 and T36. At T0, newborns with a GA ≤ 28 weeks had higher sCr levels than those with a GA > 28 weeks (Figure 1). At T36, newborns with a GA ≤ 28 weeks had lower sCr and BTP, and higher urea levels than those with a GA > 28 weeks (Figure 1). No difference was found for CysC values at T0 and T36 according to GA.

sCr levels decreased over time both in subjects with a GA ≤ 28 weeks and >28 weeks, CysC decreased only in subjects with a GA ≤ 28 weeks, urea levels decreased in both groups, whereas BTP did not significantly vary over time. CysC ranged between 0.44–2.67 mg/L at T0 and between 0.75–2.1 mg/L at T36, with values higher than 2 mg/L in 5 subjects at T0, remaining higher than this cut–off only in 1 subject at T36. eGFR values are shown in Table 5. No differences were found in newborns with a GA ≤ 28 weeks and >28 weeks calculating eGFR by the four formulas using only CysC. Conversely, eGFR values estimated by other formulas were higher in subjects born at a lower GA. 

TKV/BSA increased overtime in both groups, without differences according to GA (in subjects with a GA ≤ 28 weeks 114.44 ± 42.85 mL/m^2^ at T0 vs. 355.04 ± 156.66 mL/m^2^ at T36, *p* = 0.001; in subjects with a GA > 28 weeks 105.87 ± 26.69 mL/m^2^ at T0 vs. 330.24 ± 81.4 mL/m^2^ at T36, *p* < 0.0001).

### 3.4. Correlation Analyses

At T0, sCr values were positively correlated with CysC and BPT levels (R = 0.415, *p* = 0.01 and R = 0.274, *p* = 0.04, respectively). At T0, sCr was negatively correlated with GA (R = −0.315, *p* = 0.009), whereas both CysC and BTP were not influenced by GA. T0 levels of sCr, CysC and BTP did not correlate with anthropometric parameters, even when adjusted for GA. At T36, sCr values were positively correlated with CysC and BPT levels (R = 0.527, *p* = 0.001 and R = 0.494, *p* = 0.003, respectively). CysC and BTP were directly correlated (R = 0.531, *p* = 0.001). Neither sCr nor CysC nor BTP correlated with urea at T36. Levels of sCr, CysC, BTP and urea did not correlate with anthropometric measures at T36. The T36 levels of sCr, CysC and BTP were all directly correlated with GA (R = 0.469, *p* = 0.001; R = 0.317, *p* = 0.046; and R = 0.482, *p* = 0.002, respectively).

eGFR was negatively correlated with GA according to all formulas (*p* < 0.05), but the correlation did not persist when adjusted for weight, length, and urea levels at T36. No correlation was found between kidney volume and eGFR according to all formulas.

Postnatal kidney injury risk scores were negatively correlated with GA (R = −0.552, *p* < 0.0001).

All the anthropometric measures at T36 were inversely correlated with kidney injury risk scores. Weight at T36 was negatively correlated with both prenatal and postnatal scores (R= −0.533, *p* < 0.0001, and R = −0.337, *p* = 0.02, respectively), also when adjusted for GA. Likewise, length at T36 was negatively correlated with prenatal score (R = −0.422, *p* = 0.003), also when adjusted for GA. No correlation was found between the kidney injury risk scores and kidney volumes.

The prenatal score did not correlate with eGFR calculated with any formulas. We found a direct correlation between the postnatal score and eGFR estimated according to Schwartz 2009 (R = 0.345, *p* = 0.027) and Brion’s formulas (R = 0.312, *p* = 0.044). However, these correlations did not persist when adjusted for weight, length and BSA at T36. The correlations did not persist also when adjusted for urea levels at T36 and GA. Conversely, no significant correlations were found between the scores and eGFR according to the other formulas.

## 4. Discussion

In our study, we firstly aimed to compare the performance of CysC and BPT to traditional sCr in preterm newborns. At the 3rd day of life, CysC and BTP levels in our NICU population were comparable to the reference ranges previously reported for premature infants of similar GA [9,26], with only sCr values negatively correlated with GA. None of the biomarkers were influenced by gender or anthropometric parameters at T0. Our results are consistent with previous data by Armangil et al. in a cohort of preterm newborns with a mean GA of 32 weeks [27]. Similarly, Elmas et al. reported that CysC values measured at the 3rd day of life were independent of GA, birth weight and gender [28]. The lack of correlation we found between BTP and GA in our cohort was previously described by Filler et al. both in preterm and term newborns [29]. 

Notably, in our cohort 5 out of 71 subjects showed at T0 CysC levels higher than 2 mg/L, reported to correspond to an inulin clearance < 0.5 mL/min per kg according to Montini G et al. [30]. At T36, CysC persisted higher than this cut–off only in one infant, suggesting that the kidney function of preterm subjects tends to improve in most cases, despite potentially kidney–detrimental events during NICU staying. 

At T36, sCr and BTP levels decreased in subjects with lower GA. One could hypothesize that the lower sCr values may be the hallmark of a worse nutritional state and of a reduced muscle mass. The higher urea levels in infants with a GA ≤ 28 weeks seem to contradict this hypothesis. Therefore, it could be argued that the reduced levels of sCr and BTP found in subjects with a lower GA may indicate a better kidney function. In our cohort CysC values were independent of GA at T36. All the three biomarkers were not influenced by anthropometric measures at T36. Intuitively, because of the lower sCr and BTP levels, in the group of infants born before 28 weeks GA also the eGFR calculated by formulas using sCr or BTP (Schwartz 2009, Brion, Treiber, Benlarmi and Zappitelli–combined) were significantly higher. Interestingly, we found the unexpected result that eGFR was negatively correlated with GA, apparently indicating that subjects born at a lower GA have a better renal function at T36. However, the correlations did not persist when adjusted for weight, length, and urea levels at T36. Therefore, we may hypothesize that the confounder underlying this association is the nutritional status of preterm newborns. Indeed, in subjects born before the 28 weeks of GA, anthropometric measures at T0 were significantly lower than in those with a GA > 28 weeks, but these differences did not persist at T36. This may be probably since more premature subjects received a more aggressive and careful nutritional regimen, as suggested by the higher urea levels found in newborn with a GA ≤ 28 weeks at T36. This hypothesis may also be supported by the significantly higher percentages of subjects receiving parenteral nutrition in this group. Indeed, all newborns with GA ≤ 28 weeks received parenteral nutrition, whereas only half of the subjects with GA > 28 weeks did. 

Furthermore, we must highlight the high variability in eGFR calculated according to the nine formulas. Abitbol et al. showed in preterm and term infants that Cr–based equations consistently underestimated GFR, whereas CysC and combined equations were more consistent with reference inulin clearance studies [31].

Secondly, we aimed to assess the possible impact of pre–natal and post–natal potentially kidney-detrimental factors on kidney function of preterm newborns. In our cohort, the post–natal injury risk score was higher in newborns with lower GA, as intuitively foreseeable in more premature babies with a longer NICU stay. Therefore, we expected that subjects born at a lower GA and facing more potentially kidney-detrimental events would have an impaired kidney function at T36, as estimated by eGFR. Surprisingly, we found a direct correlation between post–natal score and eGFR estimated according to the formulas of Schwartz 2009 and Brion. However, these correlations did not persist when adjusted for weight, length and BSA. Conversely, no significant correlations were found between the score and eGFR according to the other formulas. Indeed, the Schwartz 2009 and Brion formulas rely on sCr values, that are known to be influenced by nutritional status and muscle mass. In our cohort, sCr at T36 was significantly lower in subjects with lower GA. Moreover, such correlations did not persist also when adjusted for urea levels at T36, probably for the same reasons already explained for the inverse correlation between eGFR and GA. eGFR at T36 does not seem to be influenced by prenatal factors. Moreover, neither prenatal nor post–natal factors influence kidney volumes at T36 in our cohort. 

## 5. Limitations

The post–natal variables considered as potentially kidney-detrimental are basically hallmarks of a rougher and more troubled NICU course. Therefore, they probably impair other functions of the preterm organism, and it would be difficult to disentangle the additive or synergic effects of these alterations. Moreover, due to the limited sample number, we could not assess the single contribution of all the considered risk factors on renal function. Another limit of our study is the lack of a gold standard for renal function, as it was not assessed by direct methods. Finally, we did not perform either the assessment of lean mass (for example, by bioimpedentiometry), or US and biochemical evaluation of liver function, that could have an impact on sCr production and metabolism, potentially leading to an under- or– over-estimate of its levels in preterm newborns.

## 6. Conclusions 

As almost 13 million infants worldwide are born prematurely each year, it is important to identify those at higher risk for developing chronic kidney disease due to congenital or acquired low nephron number. 

CysC seems to be a promising biomarker of renal function, as it is not influenced by GA, gender or anthropometric parameters soon after birth and by gender, anthropometric parameters and nutritional status at 36 weeks of post–menstrual age. In preterm infants, the chance to precociously detect kidney damage could allow prompt interventions that might prevent future and more serious/irreversible damages. However, its measurement is quite expensive and not widespread.

In the perspective of the best care for the preterm newborn, the potentially damaging events occurring in prenatal life have little if no impact on future kidney health. Conversely, postnatal clinical course may influence kidney function, mainly the nutritional status of preterm infants. Indeed, subjects born at a lower GA but receiving more intensive and targeted nutritional interventions may have a better renal function.

To our knowledge, this is the first study reporting a kidney-sparing effect of nutritional strategies in preterm newborns. From a clinical point of view, our findings should raise the awareness of the crucial role of nutrition, starting from the very beginning of life. 

Future studies are needed to understand which practices during the stay in NICU need to be changed to preserve future kidney function, and to evaluate which nutritional strategies would be more protective of renal health.

## Figures and Tables

**Table 1:** Items considered in prenatal (range 0–4 points) and postnatal (range 0–10 points) kidney injury risk scores (each ‘Yes’ answer accounts for 1 point).

Prenatal Kidney Injury Risk Score	Postnatal Kidney Injury Risk Score
IUGR	Born SGA
Maternal pre-eclampsia	Need for invasive ventilation
Nephrotoxic medications during pregnancy	Need for non-invasive ventilation
Positive family history for arterial hypertension	BPD
	Sepsis
	Use of nephrotoxic antimicrobic agents
	Ibuprofen for PDA
	Surgery for PDA
	NEC
	IVH

IUGR: intrauterine growth restriction, SGA: small for gestational age; BPD: bronchopulmonary dysplasia; PDA: patent ductus arteriosus; NEC: necrotizing enterocolitis; IVH: intra-ventricular hemorrhage.

**Table 2:** Clinical course data at T36, in the overall group and according to gestational age (GA).

	Overall (*n* = 53)	GA ≤ 28 w (*n* = 25)	GA > 28 w (*n* = 28)	*p*
Need for invasive ventilation (*n*, %)	30 (56.6%)	17 (68%)	13 (46.4%)	NS
Duration of invasive ventilation (days, mean ± standard deviation (SD))	8.1 ± 13.9	12.4 ± 15.9	4.4 ± 10.8	0.02
Need for non-invasive ventilation (*n*, %)	48 (90.6%)	25 (100%)	23 (82.1%)	0.02
Duration of non-invasive ventilation (days, mean ± SD)	17.5 ± 15.9	29.1 ± 15.4	7.2 ± 6.6	<0.0001
Duration of need for O2-support (days, mean ± SD)	22.8 ± 44	37.3 ± 51.7	9.9 ± 31.4	0.007
BPD (*n*, %)	14 (26.4%)	12 (48%)	2 (7.1%)	0.001
Sepsis (*n*, %)	9 (17%)	6 (24%)	3 (10.7%)	NS
Duration of antimicrobial therapy (days, mean ± SD)	17.7 ± 14.6	24.3 ± 17.2	11.8 ± 8.6	0.002
Use of nephrotoxic antibiotics (*n*, %)	50 (94.3%)	24 (96%)	26 (92.9%)	NS
PDA treated with ibuprofen (*n*, %)	19 (35.8%)	13 (52%)	6 (21.4%)	0.02
PDA treated with surgery (*n*, %)	5 (9.4%)	4 (16%)	1 (3.6%)	NS
NEC (*n*, %)	5 (9.4%)	4 (16%)	1 (3.6%)	NS
IVH (*n*, %)	12 (22.6%)	9 (36%)	3 (10.7%)	0.03
Length of NICU stay (days, mean ± SD)	65.2 ± 34.5	83 ± 35	50.7 ± 26.7	<0.0001
Parenteral nutrition (*n*, %)	40 (75.5%)	25 (100%)	15 (53.6%)	<0.0001
Duration of parenteral nutrition (days, mean ± SD)	21.2 ± 11.4	24.6 ± 13.4	15.7 ± 4.2	0.02
Prenatal kidney injury risk score	1.5 ± 0.9	1.4 ± 0.9	1.6 ± 0.9	NS
Postnatal kidney injury risk score	3.6 ± 1.6	4.5 ± 1.6	2.9 ± 1.3	<0.0001

*n*: number; BPD: bronchopulmonary dysplasia; NEC: necrotizing enterocolitis; IVH: intra-ventricular hemorrhage; NICU: neonatal intensive care unit.

**Table 3:** Anthropometric data in the overall population and in subjects with a GA≤ or >28 weeks, at T0 (3rd day of life) and T36 (gestational age 36 weeks). Data are expressed as mean ± SD (ranges).

	T0			T36		
	Overall (*n* = 71)	GA ≤ 28 w (*n* = 31)	GA > 28 w (*n* = 40)	Overall (*n* = 53)	GA ≤ 28 w (*n* = 25)	GA > 28 w (*n* = 28)
Weight (g)	1084 ± 380 (490–2236)	829 ± 215 * (490–1450)	1281 ± 363 * (580–2236)	1863.7 ± 358.3 (950–2795)	1861 ± 373 (1360–2795)	1866 ± 350 (950–2460)
Length (cm)	36.4 ± 4.1 (28–46)	33.5 ± 3.2 ** (28–40)	38.6 ± 3.4 ** (30–46)	41.8 ± 2.7 (36–48)	41.8 ± 2.5 (37–48)	41.8 ± 2.8 (36–47)
BSA (m^2^)	0.104 ± 0.024 (0.062–0.169)	0.088 ± 0.015 ^§^ (0.062–0.128)	0.117 ± 0.216 ^§^ (0.069–0.169)	0.149 ± 0.018 (0.097–0.196)	0.149 ± 0.018 (0.126–1.196)	0.148 ± 0.019 (0.097–0.180)

*, **, ^§^ *p* < 0.0001. BSA: body surface area.

**Table 4:** Biochemical data in the overall population and in subjects with GA≤ or >28 weeks, at T0 and T36.

	Overall			GA ≤ 28 w			GA > 28 w		
	T0 (*n* = 71)	T36 (*n* = 53)	*p*	T0 (*n* = 31)	T36 (*n* = 25)	*p*	T0 (*n* = 40)	T36 (*n* = 28)	*p*
sCr (mg/dL)	0.83 ± 0.22	0.30 ± 0.07	<0.0001	0.88 ± 0.22	0.27 ± 0.07	<0.0001	0.79 ± 0.22	0.33 ± 0.05	<0.0001
CysC (mg/L)	1.57 ± 0.33	1.46 ± 0.29	NS	1.62 ± 0.41	1.38 ± 0.35	0.008	1.54 ± 0.25	1.55 ± 0.19	NS
BTP (mg/L)	1.484 ± 0.464	1.299 ± 0.381	NS	1.339 ± 0.418	1.128 ± 0.306	NS	1.583 ± 0.474	1.48 ± 0.374	NS
Urea (mg/dL)	22.3 ± 1.4	9.8 ± 6.9	<0.0001	25 ± 12.9	12.5 ± 7.9	<0.0001	21.3 ± 11.3	7.36 ± 4.84	<0.0001

sCr: serum Cretinine; CysC: Cystatin C; BTP: Beta-Trace Protein.

**Table 5:** Estimated glomerular filtration rate (eGFR) values at T36 calculated by nine different formulas, in the overall population and according to GA. All eGFR are expressed in ml/min/1.73 m^2^.

	T36			
	Overall (*n* = 53)	GA ≤ 28 w (*n* = 25)	GA > 28 w (*n* = 28)	*p*
eGFR Schwartz 2009 *	59.5 ± 15.1	65.7 ± 17.1	53.2 ± 9.5	0.012
eGFR Brion *	47.5 ± 12	52.5 ± 13.6	42.5 ± 7.6	0.012
eGFR Schwartz 2012 **	51.8 ± 12	55.7 ± 15	47.6 ± 5.3	NS
eGFR Zappitelli **	51.9 ± 15.7	56.9 ± 19.6	46.3 ± 6.5	NS
eGFR Filler **	63.5 ± 18.3	69.3 ± 22.9	56.9 ± 7.6	NS
eGFR Dorum **	48.8 ± 17.2	54.3 ± 21.6	42.6 ± 6.8	NS
eGFR Treiber **	45.2 ± 16.5	51.1 ± 19.7	37.9 ± 6.4	0.04
eGFR Benlarmi ***	66.9 ± 18	75.3 ± 18	58 ± 13.4	0.005
eGFR Zappitelli–combined ^§^	80.1 ± 20.9	88.7 ± 23.8	69.4 ± 9.5	0.009

* Formulas using sCr; ** Formulas using CysC; *** Formula using BTP; ^§^ Formula combining sCr and CysC.
